# Autumn fueling behavior in passerines in relation to migratory distance and daylength

**DOI:** 10.1002/ece3.9571

**Published:** 2023-01-16

**Authors:** Elana Rae Engert, Magnus Hellström, Susanne Åkesson

**Affiliations:** ^1^ Department of Biology, Centre for Animal Movement Research Lund University Lund Sweden; ^2^ Ottenby Bird Observatory BirdLife Sweden Ottenby Sweden

**Keywords:** autumn migration, day length, fuel deposition rate, fuel load, mass increase, maximum fueling rate, songbirds

## Abstract

Songbirds have evolved diverse strategies to cope with seasonality, including long‐, medium‐, and short‐distance migration. There is some evidence that birds with a longer migration distance deposit fuel faster. However, most studies focus on long‐distance migrants. Comparisons between species with different migration distances are necessary to broaden our understanding of fueling capacity in migratory birds. We present maximum fuel deposition rates of five songbird species migrating along the southeast coast of Sweden in autumn with migration distances ranging from long (neotropical migrant) to short (partial/irruptive migrant) (Willow Warbler *Phylloscopus trochilus*, Lesser Whitethroat *Curruca curruca*, Common Chiffchaff *P. collybita*, European Robin *Erithacus rubecula*, and Blue Tit *Cyanistes caeruleus*). The birds were fed ad libitum in captivity and were exposed to either extended or natural daylength. All species ceased to increase in mass when they reached a certain fuel load, generally corresponding to migration distance, despite unlimited access to food and ample time for foraging. Blue Tits, Willow Warblers, and Lesser Whitethroats had the highest fuel deposition rates with extended daylength (19%, 20%, and 20%, respectively), and about 13% with natural daylength, which is comparable to the highest rates found in migratory songbirds in nature. European Robins and Common Chiffchaffs that winter in the temperate Mediterranean had the lowest fuel deposition rates (12% and 12% with extended daylength, respectively). Our results suggest that the long‐ and short‐distance migrants in this study have developed an extreme capacity for rapid refueling for different reasons; speedy migration to distant wintering grounds or winter survival in Scandinavia. This study contributes to our current knowledge of maximum fuel deposition rates in different species and the limitations posed by daylength. We highlight the need for future studies of species with different migration strategies in order to draw broad conclusions about fueling strategies of migratory birds.

## Graphical abstract

Maximum fueling rates were measured in songbirds migrating along the Baltic coast. The effects of daylength were investigated, and species with different migratory distances were compared.

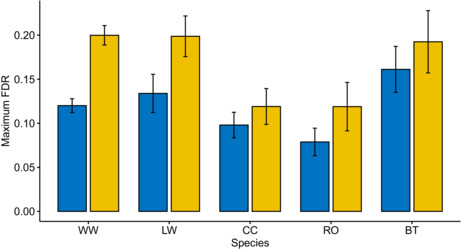

## 1 INTRODUCTION

Birds have evolved migration repeatedly and separately in many species and populations, showing remarkable diversity in migration routes and distances (Alerstam et al., 2003, Helbig, 2003). For instance, long‐distance migrants like the Willow Warbler *Phylloscopus trochilus* fly over 13,000 kilometers from their breeding grounds in eastern Russia to their tropical wintering grounds in sub‐Saharan Africa (Sokolovskis et al., 2018) while medium‐distance migrants like the European Robin *Erithacus rubecula* breed in Fennoscandia and fly roughly a quarter of that distance, wintering in the temperate climate of the Mediterranean (Fransson et al., 2008). Partial migrants, such as the Blue Tit *Cyanistes caeruleus*, may migrate only a short distance or not at all, depending on environmental conditions (Chapman et al., 2011). The migratory phenotype in songbirds involves adaptations in wing morphology, a navigation program, coordination with seasonal and diel timing, and fueling capacity, enabling birds to journey far from their natal breeding site in the autumn and to find their way back the following spring (Åkesson et al., 2021). However, these characteristics did not necessarily evolve specifically for the purpose of migration (Piersma et al., 2005). Traits that previously existed in ancestral resident species may have been taken to the extreme in long‐distance migrants. This could help explain why highly migratory species within former genus *Sylvia* (now *Sylvia* and *Curruca*) and genus *Phylloscopus* are more closely related to less migratory species than to other highly migratory species (Helbig, 2003).

One example of an adaptation that migratory birds make use of is hyperphagia, or the ability to consume and process large amounts of food in order to rapidly accumulate large fat stores (Klaassen et al., 1997; Kvist & Lindström, 2000, 2003; Lindström & Kvist, 1995). Storing high amounts of fat is vital for the survival of birds that are resident in cold climates, but it also allows nocturnal migrants to save time during migration by storing enough fuel to undertake long night‐time flights (Lindström, 2003). The speed of migration is of utmost importance to long‐distance migrants due to the vast distances that separate their breeding and wintering grounds, and the fitness benefits of arriving early (Alerstam, 2011). The most time‐consuming task during migration is not flight itself but fattening up before each leg of their journey (Hedenström & Alerstam, 1997). Consequently, the overall speed of migration depends heavily on the fueling rate that the bird can achieve at the initial staging area and at stopover sites (Karlsson et al., 2012; Lindström et al., 2019).

It is apparent that long‐distance migrants should refuel as fast as possible to maximize the speed of migration. However, there are both ecological and physiological limitations to the fuel deposition rate (FDR) and fuel load (FL) that birds can reasonably achieve (Lindström, 1991). Many birds in the wild are limited by food availability (Åkesson et al., 1995; Dänhardt & Lindström, 2001), and there may be competition for limited resources (Lindström et al., 1990). Even when resources are abundant, daylength can set limits to daily energy intake by dictating the time available for foraging (Kvist & Lindström, 2000). One must also consider foraging‐specific predation risk (Bayly, 2006), and mass‐specific predation risk which negatively affects escape probability in passerines during stopovers (Kullberg et al., 1996, 2000), and the energetic burden of flying with a heavy fuel load (Alerstam & Lindström, 1990). FDR and FL are therefore expected to be optimized with respect to migration speed, transport costs, and predation risk (Alerstam, 2011; Alerstam & Lindström, 1990; Hedenström & Alerstam, 1997; Klaassen & Lindström, 1996). According to optimal migration theory, long, medium, and short‐distance migrants likely use different strategies, with long‐distance migrants prioritizing migration speed and short‐distance migrants minimizing the mass‐specific cost of transport, metabolic costs, and predation risks (Alerstam & Lindström, 1990). Individuals migrating late in the season are also expected to have higher fueling rates than their earlier counterparts, which can be achieved by exhibiting risky foraging behaviors due to an increased time pressure and shorter daylength in autumn.

Observed differences in the fueling behavior of different species or subspecies have been attributed to differences in migration distances and routes (Berthold, 1974; Eikenaar et al., 2014; Gomez et al., 2014; Maggini & Bairlein, 2010). In a study of 213 European bird species, observed maximum fuel loads were positively correlated with migration distance (Vincze et al., 2019). Long‐distance migrants have also been shown to attain faster migration speeds than medium‐ or short‐distance migrants (Alerstam & Lindström, 1990). Direct comparisons of the maximum FDR and FL of long‐, medium‐, and short‐distance migrants using an experimental approach, however, are lacking.

It is unclear if birds regularly reach their maximum FDR in the wild, which would be possible only if food and time were not limiting factors. There are some examples of migrating birds that may maximize energy intake by exploiting energy‐rich and over‐abundant food sources, like nectar‐feeding Hummingbirds (Diamond et al., 1986) and fatty seed‐eating Bramblings *Fringilla montifringilla* (Lindström, 1990). Birds that breed at high latitudes where the sun never sets in the summer, and birds that can forage both day and night, such as waders, could potentially reach maximum daily energy intake rates due to the unlimited time for foraging each day (Klaassen et al., 2010). Many birds that breed at high latitudes do experience constant daylight in the summer (Bauchinger & Klaassen, 2005; Kvist & Lindström, 2000; Lindström, 2003), possibly during the initial staging period of refueling before departing for autumn migration. The highest recorded FDR in free‐living birds was 15% in Sanderlings, a long‐distance migratory wader (Lindström, 2003), which matches the maximum FDR recorded in a captive wader, the Common Sandpiper *Actitis hypoleucos*, with unlimited food (Kvist & Lindström, 2003). This suggests that birds can sometimes encounter ideal foraging conditions in the wild.

In theory, maximum FDR and FL can be measured by providing birds with food ad libitum and unlimited time for foraging in captivity. In this study, we measured maximum fuel deposition rates and fuel loads in passerine species with different migration distances and wintering grounds. We predicted that fuel deposition rates and fuel loads correspond to the migration distance of each species. We further expected that the effect of extended daylength on fuel deposition rate would be the largest in long‐distance migrants because they are assumed to be constrained by time available for foraging as opposed to fueling capacity. We formulated the following questions about long‐, medium‐, and short‐distance migrants in autumn. (1) How do maximum fueling rates measured in captive passerines compare to fueling rates found in nature? (2) Can differences in FDR_max_ and FL in passerine species be explained by migration distance? (3) Is FDR_max_ limited by the time available for foraging in the day?

To answer these questions, juveniles from five species of songbirds with different migration strategies and wintering grounds were captured and observed during refueling in captivity with ad libitum food under either natural or extended daylength conditions at a stopover site in southern Sweden. Information published in the Swedish Bird Ringing Atlas (Fransson et al., 2008) was used to categorize the five species as long‐, medium‐, or short‐distance migrants. Distances represent juveniles on their first autumn migration and are approximately from southern Sweden to the wintering range, and not including distance from the breeding range. Two subspecies of Willow Warblers migrate between 6000 (*P. t. trochilus*) and 10,000 km (*P. t. acredula*) to Western and Southern Africa, respectively. Lesser Whitethroats *Curruca curruca* migrate 3000–5000 km to Northeast Africa. Common Chiffchaffs *P. collybita* migrate 1000–3000 km to northern Africa and southern Europe, and European Robins migrate 500–2000 km to central and southern Europe. Blue Tits *Cyanistes caeruleus* are a partially migratory species, and populations ringed at the Baltic Sea are probably migrating short distances to other locations in southern Sweden, around 80 km (Nilsson et al., 2008).

## 2 MATERIALS AND METHODS

### 2.1 Study area

This study was carried out at Ottenby Bird Observatory (56°11′N, 16°23′E) at the southernmost point of the island of Öland, located in southeast Sweden in the Baltic Sea. The experiments ran from August 18 until November 9, 2020. Individuals of different species were caught according to their migration timing during the autumn season, starting with Willow Warblers and Lesser Whitethroats in August, and Chiffchaffs, Robins, and Blue Tits coming later in the season, respectively, until early November. In order to check for differences in early‐ and late‐migrating birds, two of the most numerous species caught at Ottenby Bird Observatory, Willow Warblers and Robins, were tested both early and late in the season, although time did not allow for a second extended daylength trail in Robins. All birds were captured as a part of the standardized ringing scheme at Ottenby Bird Observatory, using mist nets and Helgoland‐style traps in the observatory garden (Hellström et al., 2019). Birds were trapped in the morning starting 30 min before local sunrise and ending at 11 am. Each bird was ringed and the mass (nearest 0.1 g, digital scale), wing length (mm), age, and fat score (0–9 according to a visual scale for fat classification; Pettersson & Hasselquist, 1985; Sjöberg et al., 2015) was recorded. All birds in the study were juveniles in their first autumn migration and had completed their post‐juvenile molt. After ringing, birds were taken to an indoor facility at the ringing station for the remainder of the experiment. The room for keeping birds had 12 cages, a heater set to 20°C with an automatic ventilation system, and a small, east‐facing window. Each bird was kept in its own cage (50 × 30 × 40 cm) with food, drinking water, bath water, and perches.

### 2.2 Fueling experiment

Experiments lasted between 3 and 10 days, depending on how quickly individuals ceased to increase in mass. Mealworms (*Tenebrio* spp.) were provided to birds as food during all experiments. Birds were given some food immediately after capture to determine if they would eat in captivity. Birds captured with higher fat scores were given limited food for up to 3 days until they reached a fat score of 3 or less, so that they would be relatively lean and motivated to rapidly refuel at the start of the experiment (Kvist & Lindström, 2003; Schaub & Jenni, 2000). The total time in captivity before the start of the experiment ranged from half a day to 3 days, depending on the initial fat score of the bird. Birds that did not eat were released. 6 Willow Warblers (18%), 1 Lesser Whitethroat (8%), 3 Chiffchaffs (21%), 2 Robins (11%), and 0 Blue Tits were released because they did not eat in captivity.

All birds had access to food at least 20 min before the handling time so that they had the opportunity to fill their crop. Birds that had run out of food before their first weighing were documented and their FDR on the first day was excluded from the analyses. One bird that ran out of food before the first weighing was excluded entirely because it gained mass at less than half the rate of the first day, so it was not considered to be maximizing fueling rate on the second day. On the first day of the experiment, birds were weighed, and their fat score was recorded before receiving unlimited food at the end of the handling time. For each subsequent day, birds had access to food for 23 h per day, and food was removed from their cages for 1 h per day, during the handling time. The handling time was scheduled in the afternoon before sunset, which was at 17:00 for all long‐distance migrants in trials 1–4. For medium‐distance migrants in trials 5–8, it was at 16:00 due to the sun setting earlier as the season progressed. Trials 7 and 8 occurred after daylight savings so the time was recorded as 15:00. Specific information about each treatment can be found in Table 1. During this hour, the weight and fat score of each bird was recorded, and leftover food in each cage was weighed to the nearest 0.1 g. At the end of the handling time, a known amount of new food was added to each cage. The experiment ended when each bird stopped increasing in mass by more than 0.2 grams in 24 h.

**TABLE 1:** Details of each trial in a study of captive passerines at Ottenby Bird Observatory in southeast Sweden, including the trial number, daylength treatment group as natural daylength (control) or extended daylength (extended), daylength (number of hours), starting and ending date of experiment (month/day), time of handling (1 h each day), species, and sample size (*n*).

Trial	Treatment	Daylength	Date	Handling time	Species	*n*
1	Control	15.00	8/18–8/26	17:00–18:00	Willow Warbler	7
2	Extended	24.00	8/27–9/3	17:00–18:00	Lesser Whitethroat	5
2	Extended	24.00	8/27–9/3	17:00–18:00	Willow Warbler	4
3	Control	13.75	9/3–9/15	17:00–18:00	Lesser Whitethroat	5
3	Control	13.75	9/3–9/15	17:00–18:00	Willow Warbler	6
4	Extended	24.00	9/15–9/23	17:00–18:00	Willow Warbler	7
5	Control	12.00	9/26–10/9	16:00–17:00	Common Chiffchaff	5
5	Control	12.00	9/26–10/9	16:00–17:00	European Robin	6
6	Extended	24.00	10/10–10/20	16:00–17:00	Common Chiffchaff	5
6	Extended	24.00	10/10–10/20	16:00–17:00	European Robin	6
7	Control	09.50	10/26–11/3	15:00^a^–16:00^a^	Blue Tit	6
7	Control	09.50	10/26–11/3	15:00^a^–16:00^a^	European Robin	4
8	Extended	24.00	11/6–11/9	15:00^a^–16:00^a^	Blue Tit	6

^a^ Time in trials 7 and 8 was after daylight savings, shifting the local time 1 h earlier.

### 2.3 Daylength manipulation

All birds were kept in one room where daylength was controlled following Åkesson et al. (2021) with a LED lamp (Lumak Pro; 8000 lm luminous flux) that was controlled by a timer. In the control treatments, the timer was set to the natural daylength (sunrise to sunset) on the first day of the trial and was kept constant for all subsequent days of the trial. Natural light could still come through the small window, and there was some dim light entering the room from outside for about 30 min before sunrise and after sunset in the control trials. The natural daylength varied from 15 h in mid‐August to 9.5 h in late October. The daylength for each trial can be found in Table 1. Assuming that time‐minimizing migrants increase their energy intake linearly with time available for foraging (Kvist & Lindström, 2000), daylength was increased dramatically in the extended trial in order to achieve the maximum possible effect. During the extended trials, the lamp was continually kept on 24 h per day, except for about the first 15 min of the handling time, when dimming the lights was necessary to capture the birds in their cages. The time available for foraging in each trial was considered as the daylength minus the 1 h when birds were handled each day.

### 2.4 Statistical methods

All statistical tests and calculations were carried out using R version 4.0.0 and RStudio version 1.3.959 (R Core Team, 2020; RStudio Team, 2020). The lean body mass (LBM) of each bird was estimated with a linear regression analysis of body mass dependent on wing length for each of the study species from a dataset of lean birds (fat score of 0 or 1) ringed at Ottenby Bird Observatory in the years 2010–2020 (Willow Warbler, *n* = 765, Lesser Whitethroat, *n* = 359, Common Chiffchaff, *n* = 245, European Robin, *n* = 5008, Blue Tit, *n* = 309). There was a significant relationship between wing length and lean body mass for all species (Table 2). Fuel deposition rates (FDR) were calculated as the change in mass since the previous day (m_2_–m_1_), divided by LBM.

$$\mathrm{FDR}=\frac{m_{2}-m_{1}}{\mathrm{LBM}}$$

Fuel load (FL) was considered as the maximum mass that a bird reached during the experiment (*m*_max_) minus LBM, divided by LBM.

$$\mathrm{FL}=\frac{m_{\max}-\mathrm{LBM}}{\mathrm{LBM}}$$

FDR_max_ was defined as the highest FDR recorded for an individual in one 24‐h period. Early‐ and late‐migrating Willow Warblers FDR_max_ were compared using two‐sample *t*‐tests. Differences in FDR_max_ and FL between different species and daylength treatments were assessed using two‐way ANOVA. Although each of the five species can be considered as either long‐, medium‐, or short‐distance migrants, they have different migration distances and wintering grounds and they may differ in traits such as diet or diel activity patterns, so we elected to consider all possible pairwise comparisons in post hoc tests. The assumptions of linear regressions, two‐sample t‐tests, and two‐way ANOVA were determined to be fulfilled using formal tests for normality and homogeneity of variances (Shapiro–Wilks test and Levene's test).

**TABLE 2:** Linear regression results for estimating lean body mass (LBM) from wing length (“w” in regression equation) measured to the nearest millimeter in five study species ringed at Ottenby Bird Observatory, Southern Sweden between 2010 and 2020. Mean LBM, range, standard deviation (SD), and sample size (*n*) from each dataset are presented.

Species	Mean LBM (g)	Range	SD	*n*	Regression equation (LBM)	DF	*F*	*R* ^2^	*p*
Willow Warbler	7.8	6.1–10.5	0.7	765	0.17 w − 3.27	763	656.43	0.46	<.001***
Lesser Whitethroat	10.9	8.5–14.0	0.7	359	0.10 w + 4.02	357	19.16	0.05	<.001***
Common Chiffchaff	6.9	5.5–8.3	0.5	245	0.12 w − 0.02	243	219.58	0.47	<.001***
European Robin	14.7	11.3–20.5	1.0	5008	0.16 w + 2.59	5006	512.81	0.09	<.001***
Blue Tit	10.3	8.3–14.5	0.7	309	0.16 w − 0.52	307	79.3	0.20	<.001***

## 3 RESULTS

In total, 72 individuals were included in the fueling experiments, and average FDR_max_, FL, and maximum mass were calculated for each species and treatment (Table 3). In all species, mass gain was rapid at first and gradually leveled off when a specific fuel load was reached, and this fuel load was generally kept stable until they were released. One exception was Blue Tits, which started to decrease in mass after peaking on the first or second day. Many birds reached maximum values on the fat score scale, with fat deposited in the furcular hollow, flanks, and abdomen.

**TABLE 3:** Results of fueling experiments in five species of migratory passerines in southeast Sweden in autumn with natural (control) or extended daylength treatments.

Species	Treatment	*n*	Mean LBM	Mean max mass	Range	Mean FDR_max_	Range	Mean FL	Range
Willow Warbler	Control	13	8.2	11.2	9.1–12.5	0.12	0.08–0.16	0.37	0.22–0.51
	Extended	11	8.2	11.2	8.9–13.1	0.20	0.14–0.26	0.37	0.25–0.55
Lesser Whitethroat	Control	5	10.8	14.4	13.5–15.3	0.13	0.09–0.21	0.34	0.26–0.43
	Extended	5	10.7	14.4	12.6–15.8	0.20	0.16–0.29	0.35	0.18–0.47
Common Chiffchaff	Control	5	7.7	9.8	8.9–10.7	0.10	0.07–0.14	0.28	0.18–0.38
	Extended	5	7.3	9.4	7.8–10.4	0.12	0.04–0.16	0.28	0.14–0.34
European Robin	Control	10	14.4	17.5	15.8–19.8	0.08	0.02–0.19	0.22	0.11–0.36
	Extended	6	14.6	18.8	16.2–20.6	0.12	0.06–0.24	0.29	0.14–0.39
Blue Tit	Control	6	10.3	13.2	11.8–14.6	0.16	0.06–0.23	0.28	0.19–0.37
	Extended	6	9.9	12.7	12.1–13.4	0.19	0.10–0.35	0.28	0.21–0.33

*Note*: Values show mean lean body mass (LBM), mean maximum mass (heaviest mass in grams recorded for each individual), mean maximum fuel deposition rate as a proportion of LBM (FDR_max_), and mean fuel load (FL) as a proportion of LBM.

### 3.1 Fuel load

**FIGURE 1:**
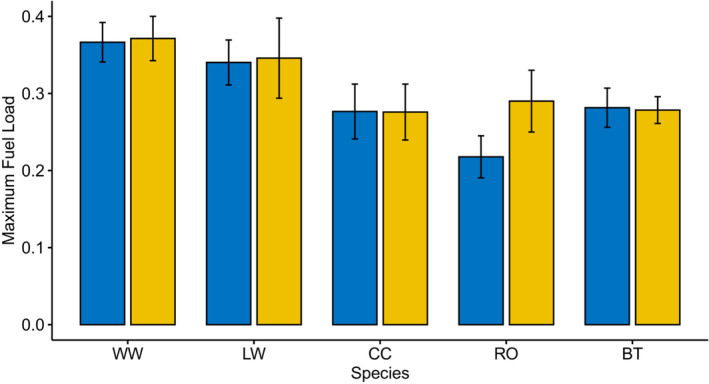
Maximum fuel load as a proportion of lean body mass in Willow Warblers (WW), Lesser Whitethroats (LW), Common Chiffchaff (CC), European Robin (RO), and Blue Tits (BT) with natural daylength (blue, 9.5–15 h) and extended daylength (yellow, 24 h) with ad lib food in captivity. Error bars show standard error.

In general, the birds reached a fuel load well above their estimated lean body mass, and mean FLs were between 0.22 in Robins and 0.37 in Willow Warblers (Figure 1). There was no significant effect of daylength treatment on FL (two‐way ANOVA, *F*_1,62_ = .70, *p* = .41), but there was a significant effect of species (*F*_4,62_ = 6.2, *p* < .001). There was no significant interaction between treatment and species (*F*_4,62_ = 0.50, *p* = .74). Willow Warblers had significantly higher fuel loads than Chiffchaffs (post hoc Tukey's HSD, *p* = .04), Robins (*p* < .001), and Blue Tits (*p* = .04). Lesser Whitethroats had significantly higher fuel loads than Robins (*p* = .05) (Table 4).

**TABLE 4:** Pairwise comparisons (Tukey's HSD) between maximum fuel load of Willow Warblers (WW), Lesser Whitethroats (LW), Common Chiffchaffs (CC), European Robins (RO), and Blue Tits (BT).

Species 1	Species 2	Diff	Lwr	Upr	Adjusted *p*	Sig
LW	WW	−0.03	−0.12	0.07	.93	
CC	WW	−0.09	−0.18	0.00	.04	*
RO	WW	−0.12	−0.20	−0.05	<.001	***
BT	WW	−0.09	−0.17	0.00	.04	*
CC	LW	−0.07	−0.18	0.04	.42	
RO	LW	−0.10	−0.20	0.00	.05	*
BT	LW	−0.06	−0.17	0.04	.44	
RO	CC	−0.03	−0.13	0.07	.89	
BT	CC	0.00	−0.10	0.11	1.00	
BT	RO	0.04	−0.06	0.13	.82	

*Note*: Difference between means (diff), lower (lwr) and upper (upr) bounds of 95% confidence intervals, adjusted *p*‐values, and significance levels (sig) are shown.

### 3.2 Maximum fuel deposition rate

**FIGURE 2:**
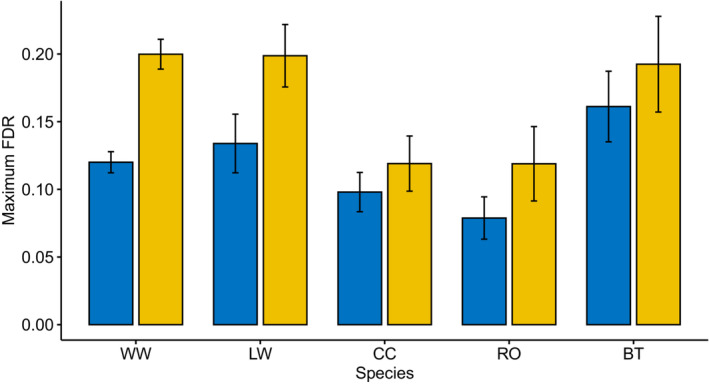
Maximum fuel deposition rate (FDR) as a proportion of lean body mass in Willow Warblers (WW), Lesser Whitethroats (LW), Common Chiffchaffs (CC), European Robins (RO), and Blue Tits (BT) with natural daylength (blue, 9.5–15 h) and extended daylength (yellow, 24 h) with ad lib food in captivity. Error bars show standard error.

The lowest average FDR_max_ was in Robins in the control group and the highest was in Willow Warblers and Lesser Whitethroats in the extended group (0.08, 0.20, and 0.20, respectively, Figure 2, Table 3). There was no significant difference in FDR_max_ between early‐ and late‐migrating Willow Warblers (two‐sample t‐test, control: t_11_ = −0.86, *p* = .41; extended: t_9_ = 0.58, *p* = .58) or European Robins (control: t_8_ = −2.06, *p* = .07) so these data were pooled for further analyses. FDR_max_ tended to be higher in the long‐ and short‐distance migrants than in the medium‐distance migrants in this study. There was a significant effect of species (*F*_4,62_ = 7.2, *p* < .001) as determined by a two‐way ANOVA. Pairwise comparisons using Tukey's HSD showed that Robins had a lower FDR_max_ than Willow Warblers (*p* < .01), Lesser Whitethroats (*p* = .01), and Blue Tits (*p* < .001), and Chiffchaffs had a lower FDR_max_ than Blue Tits (*p* = .02; Table 5).

**TABLE 5:** Pairwise comparisons (Tukey's HSD) between maximum fuel deposition rate (FDR_max_) of Willow Warblers (WW), Lesser Whitethroats (LW), Common Chiffchaffs (CC), European Robins (RO), and Blue Tits (BT).

Species 1	Species 2	Diff	Lwr	Upr	Adjusted *p*	Sig
LW	WW	0.01	−0.04	0.06	.99	
CC	WW	−0.05	−0.10	0.01	.10	
RO	WW	−0.06	−0.11	−0.02	<.01	**
BT	WW	0.02	−0.03	0.07	.79	
CC	LW	−0.06	−0.12	0.01	.09	
RO	LW	−0.07	−0.13	−0.02	.01	**
BT	LW	0.01	−0.05	0.07	.99	
RO	CC	−0.01	−0.07	0.04	.95	
BT	CC	0.07	0.01	0.13	.02	*
BT	RO	0.08	0.03	0.14	<.001	***

*Note*: Difference between means (diff), lower (lwr) and upper (upr) bounds of 95% confidence intervals, adjusted *p*‐values, and significance levels (sig) are shown.

The mean FDR_max_ was higher in the extended treatment for all five species (Table 3). There was a significant main effect of the daylength treatment (*F*_1,62_ = 19.3, *p* < .001). There was no significant interaction between treatment and species (*F*_4,62_ = 0.91, *p* = .47). Pairwise comparisons indicate a significant difference between treatments in Willow Warblers (two‐sample *t*‐test, t_24_ = −6.08, *p* < .001) and nearly significant in Lesser Whitethroats (t_8_ = −2.05, *p* = .07), but not significant in the other species (Common Chiffchaff: t_8_ = −0.84, *p* = 0.42; European Robin: t_14_ = −1.37, *p* = .19; Blue Tit: t_10_ = −0.71, *p* = .49).

## 4 DISCUSSION

In general, migration distance corresponded to FDR_max_ and FL in the birds with tropical or temperate wintering grounds, with Willow Warblers and Lesser Whitethroats having the highest average values and Robins and Chiffchaffs having the lowest. This supports the hypothesis that long‐distance migrants utilize a time‐minimizing strategy by fueling quickly and departing with higher fuel loads than medium‐distance migrants (Alerstam & Lindström, 1990; Gomez et al., 2014; Lindström, 2003). The fueling capacity of Blue Tits, however, diverged from our predictions. We must, however, use caution when interpreting our results to make broader conclusions about migratory strategies due to the small sample size, few species, and uncertainty when estimating FDR. Sample sizes and the number of species were constrained in this study mainly by the size of the bird keeping room and the number of each target species caught at Ottenby. Adverse weather sometimes precluded catching birds, and individual birds did not always adjust well to captivity. While the resulting small sample sizes may limit the robustness of our analysis, sample sizes were large enough to make meaningful comparisons between the species in this study.

Values for fuel load and fuel deposition rate in this study are based on an estimate of lean body mass for each bird, which is derived from the wing length. Explanatory power of some models used to estimate LBM were low (0.05% and 0.09% of variation in lean body mass was explained by wing length in Lesser Whitethroats and Robins, respectively, Table 2). High variation in LBM could be due to differences in fat‐free mass of birds that take different migration routes. Åkesson et al. (1992) found that robins with the same wing length that migrate over the sea are lighter overall than those migrating over land, which is probably due to a loss of muscle mass during long flights. Kelsey et al. (2019) also reported low *R* ^2^ values for Lesser Whitethroats and Robins in species‐specific models for predicting lean body mass from wing length, highlighting differences in reliability of predictions of body mass based on wing length. However, using magnetic resonance technology, they concluded that wing length is a good predictor of overall body size in songbirds. We believe that despite variation, the overall relationship between wing length and body size in the dataset provided by Ottenby Bird Observatory to be useful for estimating lean body mass of birds in this study, in the absence of more precise methods.

### 4.1 Fuel load

In migratory birds, the maximum fuel load may be regulated by the endogenous migration program and has been shown to change along the migration route with respect to magnetic cues that signal proximity to ecological barriers or the migratory destination (Fransson et al., 2001; Henshaw et al., 2008; Kullberg et al., 2003, 2007), but it can also be affected by daylength and daily activity patterns (Åkesson et al., 2021). There was no significant difference in FL between daylength treatments in this study, even though FDR_max_ was higher in the extended treatment. Individuals in the extended daylength treatment simply reached their maximum fuel load faster. In general, individuals of all species only gained weight until they reached a specific fuel load, which they maintained until they were released.

The long‐distance migrants, Willow Warblers and Lesser Whitethroats, had the highest fuel loads of 34%–37%, while the medium‐distance migrants had fuel loads of 22%–29%, which is comparable to findings in other studies of birds not immediately before an ecological barrier (Alerstam & Lindström, 1990). Blue Tits, the short‐distance migrants in this study, gained 28% of their lean body mass on average. This amount of stored fuel in a short‐distance migrant such as the Blue Tit would not be necessary for their anticipated migration distance and demands an alternate explanation. As Blue Tits were captured in late October and November, they may have already begun winter fattening, an adaptive behavior in passerines that winter in cold climates (Pravosudov & Grubb, 1997).

### 4.2 Fuel deposition rate

Overall, FDR_max_ was higher with extended daylength, regardless of the migration distance for each species. One explanation is that these species are limited by the time available for digesting and processing food, even when food is abundant. Although we found no significant interaction, this pattern seemed to be stronger in some species than in others. Within species, FDR_max_ was found to be significantly or marginally higher in the extended daylength treatment in long‐distance migrants, Willow Warblers and Lesser Whitethroats (Figure 2). This suggests that the time available for foraging, or daylength, may be an important factor controlling the FDR, and therefore the speed of migration, especially for the longer distance migrants in this study. If this is a general pattern in long‐distance migrant passerines, the implication is to migrate as early as possible in the season in autumn to maximize daylength and therefore the time available for foraging along the route. By optimizing migration speed, migrating birds could benefit from arriving early to the wintering grounds if they compete with other migratory birds for winter territories and resources (Salewski et al., 2002).

While photoperiod may play a role in regulating the annual schedule in migratory birds with respect to fuel deposition and migratory activity, the short duration of the experiment and the abrupt change in daylength makes it unlikely that birds perceived a seasonal change to summer (Åkesson et al., 2021; Müller et al., 2018). It has been shown that nocturnally migrating songbirds continue to initiate migratory restlessness and increase fuel deposition seasonally even in the absence of photoperiodic cues (Gwinner, 1996; Maggini & Bairlein, 2010). However, even abrupt changes in daylength can alter the timing and duration of migratory restlessness in migratory birds and can affect nocturnal and diurnal migrants differently (Åkesson et al., 2021). In the absence of activity and metabolic data for the duration of the experiment, it is impossible to ascertain if and to what extent the fuel deposition rate and fuel load were altered by behavior or physiology between treatments and species. Based on previous studies, we assume that nocturnally migratory birds with low fuel stores reduce their migratory activity levels when presented with abundant food (Klaassen & Biebach, 1994), but this may not hold true for diurnal migrants (Åkesson et al., 2021). Ideally, one would measure activity, energy expenditure, and fuel deposition rate in tandem to disentangle the effects of increased daylength on behavior, physiology, and foraging capacity.

The birds in this study generally were motivated to feed at high capacity and gained weight at unprecedented rates. Both long‐distance migrants in this study, Willow Warblers and Lesser Whitethroats, had an FDR_max_ of 12% and 13% on average in the control treatment, respectively, which is comparable to maximum rates found in long‐distance migrants in nature (Lindström, 2003), in supplemental feeding experiments (Bayly, 2006), and in captivity with natural daylength (Kvist & Lindström, 2000; Table 6). Taken together, this is an indication that 13% may be a good estimate for FDR_max_ in long‐distance migrant passerines when they are not limited by food availability in the wild. However, this may not be a common occurrence given that fuel deposition rates higher than 10% are seldom recorded in wild passerines or waders (Lindström, 2003). Average FDR_max_ in the extended trials for long‐ and short‐distance migrants was about 20% of lean body mass per day, which is higher than FDR_max_ previously measured in captive waders with round‐the‐clock access to food (Table 6). Given that FDR_max_ is negatively correlated with body mass and passerines are generally much smaller than waders, we can expect that passerines should be able to achieve higher FDR_max_ than most waders (Lindström, 2003).

**TABLE 6:** Average (avg) and individual (ind) maximum daily fuel deposition rates, calculated as the change in body mass per day as a proportion of lean body mass (LBM) measured in different studies with free‐living migratory waders (W) and passerines (P) with natural food sources and supplemented food, as well as captive migrants with natural daylength (in the case of average values, daylengths throughout the study are represented), and extended foraging time of 23 or 24 h per day.

Species	Latin name	Group	LBM (g)	FDR (avg)	FDR (ind)	References
Free‐living, natural conditions						
Willow Warbler	*Phylloscopus trochilus*	P	8.0	0.05		Williamson and Butterfield (1954)
European Robin	*Erithacus rubecula*	P	14.0	0.03		Dänhardt and Lindström (2001)
Bluethroat	*Luscinia svecica*	P	16.1	0.01		Lindström and Alerstam (1992)
Common Sandpiper	*Actitis hypoleucos*	W	45.0	0.03		Brown (1974)
Red Knot	*Calidris canutus*	W	93.6	0.05		Buxton (1989)
Free‐living, supplemental food						
European Reed Warbler	*Acrocephalus scirpaceus*	P	10.0		0.13	Bayly (2006)
Common Whitethroat	*Curruca communis*	P	13.5	0.10		Fransson (1998)
European Robin	*Erithacus rubecula*	P	14.0	0.05	0.09	Dänhardt and Lindström (2001)
Bluethroat	*Luscinia svecica*	P	16.1		0.05	Lindström and Alerstam (1992)
Northern Wheatear	*Oenanthe oenanthe* ssp. *oenanthe*	P	22.7	0.11		Delingat et al. (2006)
Northern Wheatear	*Oenanthe oenanthe* ssp. *leucorhoa*	P	22.7	0.13		Delingat et al. (2006)
Captive, natural foraging time						
Thrush Nightingale	*Luscinia luscinia*	P	21.6	0.09	0.15	Kvist and Lindström (2000)^b^
Willow Warbler	*Phylloscopus trochilus*	P	8.2	0.12	0.16	^a^
Lesser Whitethroat	*Curruca curruca*	P	10.8	0.13	0.21	^a^
Common Chiffchaff	*Phylloscopus collybita*	P	7.7	0.10	0.14	^a^
European Robin	*Erithacus rebecula*	P	14.4	0.08	0.19	^a^
Blue Tit	*Cyanistes caeruleus*	P	10.3	0.16	0.23	^a^
Captive, extended foraging time						
Thrush Nightingale	*Luscinia luscinia*	P	22.2	0.16	0.19	Kvist and Lindström (2000)^b^
Willow Warbler	*Phylloscopus trochilus*	P	8.2	0.20	0.26	^a^
Lesser Whitethroat	*Curruca curruca*	P	10.7	0.20	0.29	^a^
Common Chiffchaff	*Phylloscopus collybita*	P	7.3	0.12	0.16	^a^
European Robin	*Erithacus rebecula*	P	14.6	0.12	0.24	^a^
Blue Tit	*Cyanistes caeruleus*	P	9.9	0.19	0.35	^a^
Common Sandpiper	*Actitis hypoleucos*	W	38.0	0.15		Kvist and Lindström (2003)
Red Knot	*Calidris canutus*	W	103.0	0.13		Kvist and Lindström (2003)

^a^ Data collected in this study, autumn 2020.

^b^ Calculated from fueling data in Kvist and Lindström (2000) from single day greatest mass change with 10–14 h (natural) or 23 h (extended) foraging time; mass change may include mass of stomach contents in extended foraging time group.

Blue Tits, which are short‐distance migrants, had a significantly higher fuel deposition rate than Chiffchaffs and Robins, two medium‐distance migrants, and were comparable to the long‐distance migrants, Willow Warblers and Lesser Whitethroats. The high FDR of Blue Tits was an unexpected result, but there are logical explanations. Blue Tits are partial migrants that winter at northern latitudes, and they are exposed to food scarcity and hypothermia in the winter (Nord et al., 2009). Small songbirds that winter at northern latitudes are known to spontaneously increase in mass during the non‐breeding season (Blem, 2000), much like birds in migratory disposition, and Blue Tits with access to supplemental food in the wild deposit more fuel than in natural conditions (Broggi et al., 2021). A high FDR may be an adaptation for exploiting unpredictable food sources in the winter and for making the most of the shorter daylengths that they experience in the north (Alerstam et al., 2003). The fuel deposition rates in Blue Tits were similar to those measured in long‐distance migrants, which suggests that energy requirements associated with wintering at northern latitudes make high FDR a favorable adaptation, just like it is for long‐distance migrants (Broggi et al., 2021; Lindström, 2003). The two energy‐demanding activities may require a high capacity for fueling and could represent two separate selection pressures that influence the same physiological adaptation. This reflects the energy tradeoff between migration and residency in birds that breed at northern latitudes and may help explain why different strategies are maintained by evolution (Alerstam et al., 2003). The high FDR of Blue Tits could also be an indication that partial migrants and residents at northern latitudes are pre‐adapted to evolve long‐distance migration, or vice‐versa, because of their high fueling capacity (Berthold, 1988).

Medium‐distance migrants winter in temperate climates, and they spend less energy on locomotion overall than long‐distance migrants due to their shorter total migration distance. They are also expected to experience less of a time constraint to reach wintering grounds and can therefore afford to migrate slower with lower transport costs (Alerstam & Lindström, 1990). Medium‐distance migrants may not need to have as high fueling capacity as long‐distance and short‐distance migrants, and it may be advantageous to have relatively low fueling rates and fuel loads in order to save energy. This could explain the relatively low fueling rates and fuel loads found in medium‐distance migrants, even when food and daylength were not limiting factors.

## 5 CONCLUSIONS

The birds in this study had some of the highest maximum fuel deposition rates ever reported in long‐ and short‐distance migrating songbirds and were highest with extended daylength (i.e., 20% for Willow Warblers and Lesser Whitethroats with extended daylength). With natural daylength, Willow Warblers and Lesser Whitethroats, both long‐distance migrants, had an average FDR_max_ of 12%–13%, a value that is rarely recorded in nature (Lindström, 2003). This indicates that free‐living migratory birds are limited by different ecological factors such as food availability, competition, predation risk, time available for foraging, or a combination of those. Fueling rates and fuel loads are important factors that contribute to the overall speed of migration in long‐distance migrants, but they may also be instrumental in winter survival for those species that remain at northern latitudes. Free‐living birds may exploit abundant natural or anthropogenic food sources (i.e., bird feeders) at stopovers or the wintering grounds and it is important to understand the upper limit to fueling rates in species with different migration and life‐history strategies. This study highlights the importance of comparing species with different migration strategies and including partial migrants when investigating the fueling behavior of migratory birds. As this study was conducted in the autumn with only juvenile birds, it would be worthwhile to measure FDR_max_ in the spring, include adult birds, and compare males and females in future studies. Some factors that contribute to uncertainty when estimating FDR from daily mass changes are the possibility for concurrent changes in water mass, muscle mass, and mass of contents of the digestive tract. However, magnetic resonance technology can measure changes in body composition more precisely. Future studies using advanced methods such as magnetic resonance technology to measure fuel deposition rate including a range of bird species are needed in order to draw broad conclusions and compare the fueling capacities of long‐, medium‐, and short‐distance migrants.

## AUTHOR CONTRIBUTIONS

**Elana Rae Engert:** Conceptualization (equal); formal analysis (lead); investigation (lead); methodology (equal); visualization (lead); writing – original draft (lead); writing – review and editing (lead). **Magnus Hellström:** Conceptualization (supporting); investigation (supporting); methodology (supporting); project administration (supporting); resources (equal); supervision (supporting); writing – original draft (supporting); writing – review and editing (supporting). **Susanne Åkesson:** Conceptualization (equal); formal analysis (supporting); funding acquisition (lead); investigation (supporting); methodology (equal); project administration (equal); resources (equal); supervision (lead); visualization (supporting); writing – original draft (supporting); writing – review and editing (supporting).

## Data Availability

The data that support the findings of this study are openly available in Dryad at https://doi.org/10.5061/dryad.b2rbnzsk9.
